# Oral Health Knowledge, Attitude, and Approaches of Pre-Primary and Primary School Teachers in Mumbai, India

**DOI:** 10.1155/2016/5967427

**Published:** 2016-02-29

**Authors:** Ankita Mota, Kunal C. Oswal, Dipti A. Sajnani, Anand K. Sajnani

**Affiliations:** ^1^Mota's Multispeciality Dental Clinic, Shop No. 4, Batatawala Mansion Near Ganesh Galli, Dr. Babasaheb Ambedkar Road, Lalbaug, Mumbai 400012, India; ^2^Terna Dental College, Plot No. 12, Sector 22, Opposite Nerul Railway Station, Nerul West, Navi, Mumbai 400706, India; ^3^Zircon Dental Centre, Villa No. 36, Opposite Wakra Hospital Gate No. 4, P.O. Box., 1941, Wakra, Qatar; ^4^KIMS Qatar Medical Centre, Abdulrahman Bin Jassim Al Thani Street, P.O. Box. 82125, Wakra, Qatar

## Abstract

*Background*. School teachers have an internationally recognized potential role in school-based dental education and considerable importance has therefore been attributed to their dental knowledge. The objectives of this study were to determine the oral health related knowledge, attitudes, and approaches of pre-primary and primary school teachers in the city of Mumbai.* Methods*. The descriptive cross-sectional study was conducted in the suburban regions of Mumbai using a self-administered questionnaire and involved 511 teachers.* Results*. Teachers demonstrated inappropriate or incomplete knowledge regarding children's oral health. Only 53.2% knew that an individual has two sets of dentition. Moreover, only 45.4% of the teachers knew that a primary dentition consists of 20 teeth. Only 56.9% of the teachers asked their children to clean their mouth after snacking during school hours. 45.0% of the teachers were unaware of fluoridated tooth pastes whilst 78.9% of them were unaware of school water fluoridation programmes. Also, 54.8% of the teachers never discussed the oral health of children with their parents during parents meet.* Conclusions*. The studied school teachers demonstrated incomplete oral health knowledge, inappropriate oral practices, and unfavourable approaches to children's oral health. There is a definite and immediate need for organized training of school teachers on basic oral health knowledge.

## 1. Introduction

The importance of imparting lessons on hygiene to infants and pre-school children had been recognized as early as 1878 [1, 2]. The responsibility of training these children is by far the most difficult, making it imperative to select teachers with special qualifications and training. There is increasing recognition in both the scientific and social community, of the tremendous influence which a school teacher has not only in encouraging good health habits, but also in promoting overall development [3]. Over the years, schools have initiated their own health programs depending upon the health status of their children. However, very few of them actually focus on oral health promotion.

Pre-primary and primary schools have a great potential for influencing the health behavior of the child [4–7]. Children spend considerable time in school especially during the age when their habits are being formed. Hence the role of teachers during these development stages of the child is critical. It is now established that school teachers have an internationally recognized potential role in school-based dental education and considerable importance has therefore been attributed to their dental knowledge [8].

Numerous studies conducted worldwide have demonstrated the attitude, knowledge, and willingness of school teachers to promote oral health amongst their pupils [5–15]. Surveys conducted in Minnesota, USA, among future school teachers [9] and in Michigan, USA, among elementary school teachers [10] suggested that oral health knowledge of teachers was often inadequate and inaccurate. They were ill-informed and held inconsistent opinions about basic oral health related concept. Likewise, a study conducted among government primary school teachers in a rural part of India concluded that oral health knowledge was lacking among the teachers [6]. However, studies from Romania, China, and Saudi Arabia have reported positive attitudes among school teachers towards school based dental health education and a willingness to be involved in oral health promotion [5, 7, 12, 13]. Likewise, a study involving Arab school teachers in northern Israel demonstrated positive levels of dental knowledge and attitudes amongst the group and also highlighted the fact that the teacher's main reported source of knowledge was the dental office [8]. Kuwaiti school teachers revealed a higher level of dental knowledge than those of children's parents. Teachers reported a positive attitude towards the prevention of dental diseases [11].

Depending on local infrastructure and available resources, various approaches have been adopted by schools to promote oral health education. Some schools have incorporated a number of initiatives simultaneously while others have built on an existing established system of good practice [16]. In China, oral health education has been effectively integrated into the school curriculum through puppet shows, models, and flannel graphs. Also, parents are imparted health education through special programs [17]. Likewise, in Denmark, children's oral status is constantly monitored and assessed; and special attention is given to high-risks groups. Administration of topical fluoride is a regular feature and there is an interdisciplinary collaboration of nurses, school teachers, and day-care centers to prioritize oral health [18].

In India, the National Oral Health Care Programme, which is a project of Director General of Health Services (DGHS) and Ministry of Health and Family Welfare, was initiated in 1998. The programme aims to achieve optimal oral health for all by the year 2020. Amongst the numerous policies developed by the programme, one involved training of health workers and school teachers in different parts of the country. So far, 13 training programmes have been conducted in different parts of the country but none have included the city of Mumbai [19].

Mumbai, India's financial capital, is also the most populous city in the country and the fourth most populous city in the world, with a total metropolitan area population of approximately 20.5 million [20–22]. There are 4864 registered primary and pre-primary schools in the city which are either Government aided or private or run by the Municipal Department with over 91,548 teachers [23]. However, till date no data exists on the oral health attitude and awareness of these teachers who impart education to children. The primary mode of instruction in these schools is either English or Marathi (local state language). Thus, the objectives of this study were to determine the oral health related knowledge, attitudes and approaches of pre-primary and primary school teachers (both in English and Marathi medium schools) in the city of Mumbai.

## 2. Materials and Methods

The descriptive cross-sectional study conducted in the suburban regions of Mumbai between November 2010 and April 2011 involved 511 teachers. The schools were selected from the list of schools obtained from local authorities and the Planning Commission of India (2012) [23] using a stratified cluster sampling method. Stratification was done on the basis of the mode of teaching used. All these schools were numbered and schools were then selected according to a list of random numbers. Teachers who were absent on the day of data collection for any reason such as sick leaves were excluded from the study. However, the number of absentees was small and had no effect on the outcome of the study.

The strength of teachers in the school ranged from 10 to 25. A total of 30 schools were selected in each group. Once selected, the entire cluster of teacher present during the survey period was used. The study's sample size was calculated using *N* = 4*pq*/*d* ^2^ where *N* is sample size, *p* is prevalence, *q* = 1 − *p*, and *d* is estimated difference. There were no prior prevalence studies to determine the school teachers' perception of oral health in the population of study, and hence a conservative estimate of *p* = 50% was estimated. The total sample size required was 400 (200 in each group).

The research instrument was primarily a closed ended questionnaire that was pretested among 50 teachers in a pilot study in similar environments. The 26-item questionnaire sought information on sociodemographics, oral health related knowledge, practices, and awareness of school teachers and their approaches towards oral health of school children. The options for closed ended questions were obtained from relevant literature and an additional option of “others (please specify)” was included to enable the teachers to freely decide on a response [10, 24]. Some closed questions required dichotomous yes or no responses. The questionnaire was administered in English; however, any respondent who failed to comprehend the meaning of any of the questions due to language barrier was given sufficient explanation by one of the authors to be able to respond to the questions fairly. After the questionnaires were collected from the respondents, a presentation was made on the basic aspects of oral hygiene.

Permission to carry out the study was obtained from all the selected school principals, administrators and management. Informed consent was obtained from the school teachers. All the participants were assured of the confidentiality of their responses. The statistical package for social sciences (SPSS) version 13.0 for Windows was used for the analysis of data collected. The analysis was done using chi-square test (*p* < 0.05). This study was approved by the Ethics committee of Terna Dental College, Navi Mumbai, India.

## 3. Results

The demographic characteristics of all 511 teachers who participated in the study are presented in Table 1. Out of the 30 schools in each group, 25 (83.3%) from the English medium and 18 (60%) from the Marathi medium responded. Most teachers had good oral hygiene practices—94.8% of them used a tooth brush as a cleaning aid and 92.6% of them used a tooth paste along with it (Table 2). The remaining 2.2% teachers used a tooth brush only without any cleaning agents. Most teachers (76.3%) brushed their teeth twice in a day (*p* = 0.00). Also, teachers from both groups (English and Marathi) demonstrated the use of potentially traumatic and inappropriate accessory tooth cleaning aids including toothpicks (*p* = 0.02) (Table 2).

Respondents from both groups had inappropriate or incomplete knowledge regarding children's oral health. A little more than half of the respondents (53.2%) knew that an individual has two sets of dentition—primary and permanent. However, 76.3% of them accepted that primary dentition is an important aspect of the health of the child (Table 3).

Out of the 511 teachers who participated, 453 (88.7%) believed that a child should brush twice a day. Majority of the teachers (71.1%) responded that they were aware of the brushing techniques of the children (*p* = 0.04). However, they responded inconsistently to the types of brushing techniques with 18.0% suggesting horizontal technique, 14.7% suggesting vertical technique, whilst 41.3% recommending circular motions as an ideal technique for brushing children's teeth (*p* = 0.04) (Table 4).

Most teachers demonstrated inappropriate approaches to monitoring oral health of children whilst in school and lack of awareness of effects of fluoride (Table 4). Only 56.9% of the teachers asked their children to clean their mouth after snacking during school hours. Moreover, 54.8% of the teachers never discussed the oral health of children with their parents during parents' meetings meet despite the fact that 74.0% (*p* = 0.036) of them agreed that oral health affects general health (Table 4).

Primary school teachers in English medium had a significantly higher level of education compared to pre-primary school teachers (*p* = 0.05). Likewise, primary school teachers in Marathi medium school demonstrated a higher level of education than their counterparts in pre-primary school (*p* = 0.00) (Table 5). Primary school teachers in English medium school had a better knowledge of “the dentition an individual has” when compared to their colleagues in pre-primary school (*p* = 0.05). The primary school teachers in Marathi medium school demonstrated a similar trend (*p* = 0.05) (Table 5).

## 4. Discussion

The data on school teachers were collected by means of self-administered questionnaires mainly because of practical and economic reasons. There was a low response rate of 25 English and 18 Marathi schools from the 30 schools shortlisted in either group. The primary reasons for these finding were the lack of communication on part of the school and failure to obtain approval from the concerned authorities.

Use of self-administered questionnaires in gathering health related information has its limitations in identifying cause-effect relationship [25]. Nevertheless, all efforts were made to minimize the adverse response biases by maximizing confidentiality during data collection to influence the degree of frankness and ensure understanding of the given instructions, types of items and situations [14, 26]. Moreover, this method of collecting data has been tested previously and has shown adequate reliability [5, 8, 10, 13].

Toothbrushes and toothpastes have been universally accepted as tools for daily oral hygiene maintenance. However, the uses of these aids have been limited in some communities due to traditional and cultural practices as well as lack of awareness of the benefits of these tools. In the present study, a vast majority of teachers used tooth brush as a tooth cleaning medium and tooth paste as a tooth cleaning material. However, a small percentage of them did use harmful media including tooth powder and misheri (smokeless tobacco product generally prepared by baking tobacco on a hot metal plate until toasted or partially burnt). The reason for this finding could not be ascertained as it was not an objective of the present study; however, one possible explanation could be the influence of family traditions in routine practices.

Dental floss and other interdental cleaners are recognized as an important part of dental hygiene and are required daily to remove plaque and other particulate matter from between the teeth and gingival line [15, 27, 28]. The percentage of teachers using dental floss as an oral hygiene maintenance tool is very low and is similar to figures reported for Nigerian school teachers (8.5%) [15]. Interestingly, though, many of the teachers did use mouth wash as an accessory tooth cleaning aid. Whilst the reason behind this discrepancy could not be ascertained, one plausible explanation could be the active promotion of mouth washes in visual media and newspapers.

Various schools of thought are divided on the recommended interval between dental check-ups. The frequency of dental visit should be determined specifically for each patient depending on the level of disease and the specific risk factors. Nevertheless, preventive annual dental visit is widely considered a standard oral health practice. In the current study, almost half of the teachers did not have a routine dental checkup done in the past year. Many teachers visited a dentist primarily because they had pain in their oral region or they could recognize a symptom. This finding is consistent with studies done elsewhere [15].

Teachers in both groups (English and Marathi) exhibited lack of basic knowledge on primary and permanent teeth. Only a little more than half of the respondents confirmed that humans have two types of dentition—primary and permanent. Majority of them (40.1%) thought that a human being has only set of dentition—permanent. Moreover, only 45.4% of the teachers knew that primary dentition consists of 20 teeth. Furthermore, primary school teachers in both English and Marathi medium schools demonstrated a better knowledge of “the dentition an individual has” when compared to their counterparts in pre-primary school. This finding could be attributed to the fact that primary school teachers had a statistically significant higher level of education when compared to the colleagues in pre-primary school. Also, most of the primary school teachers had more teaching experience when compared to their counterparts in pre-primary school. Interestingly, though, 76.3% believed that primary teeth is important to general health. This finding has probably been overreported considering the fact that the teachers had poor basic knowledge on primary teeth. Also, the reasons for a child's first visit to a dentist had been inconsistently reported. This is a clear indication of the lack of awareness and knowledge on the basic aspects of children's oral health.

Majority of the teachers (88.7%) believed that a child should brush at least twice a day and expected exceptional oral hygiene practices from children. However, the teachers themselves exhibited moderate oral hygiene practices. Another 10% believed that children should brush their teeth thrice a day to maintain adequate oral hygiene. Likewise, though 71.1% of the teachers claimed to be aware of the brushing techniques for children, the techniques were inconsistently reported by them.

Fluoride containing compounds have been used in preventing incipient carious lesions since the early 1900s [29]. Optimal water fluoridation has been recognized as the single most cost-effective public health measure known to science for preventing tooth decay [8, 25]. Hence it was deemed essential to determine the knowledge and attitudes of school teachers towards the subject of fluorides. This knowledge was unknown to teachers surveyed in this study; a finding similar to other studies [8]. Whilst less than half of the teachers knew about fluoridated toothpastes, only 7.2% aware of the school water fluoridation programs.

Traditionally, school teachers have been considered as important primary agents of socialization and have been shown to influence the future knowledge, attitude and behaviour of school children [8, 30, 31]. In some studies teachers have demonstrated willingness to participate in oral health education [5, 8, 11–13] whilst in others this role has not been readily accepted [32]. In the present study, teachers reported lack of interest for roles such as “advising the children to clean their mouth after snacking” and “discussing the oral hygiene status with their parents.”

The advantages of using school personnel as oral health promoters for children are manifold. Teachers have the potential for reaching all the children and establish continuity in the instructions. Further, they can integrate oral health promotion with other activities and the entire process would be inexpensive [5, 11]. A possible disadvantage could, however, be that the teachers may not have an adequate background for providing health education [5, 11] as was also observed in the current study [10, 15]. Anecdotal evidence highlights various practical limitations for teachers to improve their oral health knowledge that also reduces their motivation to provide oral health education in schools. These primarily include lack of support for example, literature resources, and lack of reinforcements from professionals, in terms of supervisory visits, seminars, continuing education, symposiums, and workshops [14]. The present study is concordant with this finding as vast majority of teachers (81.2%) had not received any formal training on oral health education and knowledge.

It is difficult to ascertain the impact of teachers' inappropriate oral health practices and attitude on children's oral health. Whilst it is normally perceived that an individual cannot give what he does not have, there are many people who teach good practices despite the fact that they still indulge in poor practices themselves. Studies have suggested that one can have the tendency to hold high standards for others while performing morally suspect behaviours themselves [15, 33–35].

Nevertheless, the concept of using school teachers for frequent oral health education has been found to be more feasible and effective than infrequent dental health education by professionals [36]. Moreover, a teacher may assess the child's performance fairly frequently and applaud the child for his/her improved oral health. This in itself is an encouragement since it is delivered by a teacher for whom the child has respect and regard [36]. Reinforcement through repeated oral health education session has shown to induce significant improvement in the knowledge of oral health practices and reduction in plaque index scores for school children [37].

The dissemination of scientific information across a given population has always been influenced by ethnic, political, social, and cultural values. Oral hygiene education is no exception. Whilst the incomplete and inaccurate oral health knowledge exhibited by teachers in the current study cannot be justified, an important aspect that may be considered for this alarming finding could be the amount of time, resources, and finances spent by the government and official authorities. Moreover, the role of dental professionals in such scenarios cannot be ignored. In a city like Mumbai, which has adequate dental workforce, the role of dental professionals in training and supervision of teachers in the area of oral health may provide a suitable and practical option.

Unfortunately, oral health is given last priority by policy makers in India. The relevant authorities are inadequately informed about the burden of dental problems and its association with systemic health [19]. Another drawback is that health per se is a subject of the particular state government and not the central government. Most states in India, like elsewhere in the world, are suffering from financial burden even for subsistence let alone providing quality health care. Consequently, the health care, particularly oral health, is looked after by the private sector and individual practices, including non-formal medical facilities, making the treatment costs for oral diseases enormously expensive. Further, India lacks experts in dental public health and the curriculum for graduation is outmoded with very little importance to prevention. Subsequently, dental graduates are unable to perceive the importance of preventive dental programmes.

Across the world, most health economics are struggling with cut backs. Implementing oral health promotion and awareness programs in such a scenario is difficult and expensive to achieve. Support from the private healthcare sector is one way to achieve the target. Another possible way would be to run the programme via a not-for-profit organization. Various interventional programs targeting the school teachers as well as students have been implemented in different countries. A feasible approach for the city of Mumbai would be to create a special committee or council which would include representatives from the state dental council, professors in dental schools, dental assistants, school teachers, physical education instructors as well as textbook publishers [38]. The council would be responsible for identifying multiple resources of good dental health teaching material for distribution to schools. It would promote the entire school health education movement and press for better health training of teachers and other school health personnel in their training institutions and on the job.

In spite of the considerably large sample size and the positive response rate, the study is not without drawbacks. The questionnaire was administered in English and while that would not be a cause for concern for the teachers in English medium schools, some teachers from the Marathi medium schools may have had difficulties understanding a few questions. Further, since the questions are standardized, some respondents may have misinterpreted them. Nevertheless, one author was always present at the time the teachers responded to the questions to help them with translation and/or interpretation if required. Further, since the questionnaire was personally administered, a rapport could be established with the respondents and a high response rate was achieved.

## 5. Conclusions

School teachers in both English and Marathi medium schools in Mumbai demonstrated incomplete oral health knowledge, inappropriate oral practices, and unfavourable approaches to children's oral health. There is a definite and immediate need of organized training of pre-primary and primary school teachers on basic oral health knowledge.

## Figures and Tables

**Table 1:** Demographics of 511 school teachers who participated in the study.

Demographics		English (%)	Marathi (%)	Total (%)
Age	<24 years	28 (5.5)	11 (2.1)	39 (7.6)
	25–39 years	165 (32.3)	49 (9.6)	214 (41.9)
	40–59 years	102 (20.0)	153 (29.9)	255 (49.9)
	60 years and above	2 (0.4)	1 (0.2)	3 (0.6)

Gender	Male	21 (4.1)	51 (10.0)	72 (14.1)
	Female	276 (54.0)	163 (31.9)	439 (85.9)

Education	School	8 (1.6)	19 (3.7)	27 (5.3)
	Higher secondary school	66 (12.9)	42 (8.2)	108 (21.1)
	Graduation	184 (36.0)	129 (25.2)	313 (61.2)
	After graduation	39 (7.6)	24 (4.7)	63 (12.3)

Teaching	Pre-primary school	63 (12.3)	24 (4.7)	87 (17.0)
	Primary	234 (45.8)	190 (37.2)	424 (83.0)

Teaching experience	<1 year	35 (6.8)	7 (1.4)	42 (8.2)
	1–5 years	100 (19.6)	33 (6.4)	133 (26.0)
	>5 years	162 (31.7)	174 (34.1)	336 (65.8)

Marital status	Unmarried	66 (12.9)	30 (5.9)	96 (18.8)
	Married	231 (45.2)	184 (36.0)	415 (81.2)

Children	Yes	212 (41.5)	184 (36.0)	396 (77.5)
	No	85 (16.6)	30 (5.9)	115 (22.5)

**Table 2:** Knowledge and practices of school teachers regarding personal oral health.

Knowledge and practices		English (%)	Marathi (%)	Total (%)	Chi square test
Tooth cleaning medium	Tooth brush	286 (56.0)	198 (38.8)	484 (94.8)	*χ* ^2^(2) = 5.9, *p* = 0.52
	Finger	9 (1.8)	16 (3.1)	25 (4.9)	

Tooth cleaning material	Toothpaste	281 (55.0)	192 (37.6)	473 (92.6)	*χ* ^2^(3) = 7.13, *p* = 0.68
	Toothpowder	16 (3.1)	17 (3.3)	33 (6.4)	
	Misheri	0 (0)	2 (0.4)	2 (0.4)	
	Other	0 (0)	2 (0.4)	2 (0.4)	

Number of times teeth cleaned	Once	72 (14.1)	23 (4.5)	95 (18.6)	*χ* ^2^(3) = 19.3, *p* = 0.00
	Twice	209 (40.9)	181 (35.4)	390 (76.3)	
	Thrice or more	11 (2.2)	4 (0.8)	15 (3.0)	

Accessory tooth cleaning aids	Floss	14 (2.7)	7 (1.4)	21 (4.1)	*χ* ^2^(4) = 11.04, *p* = 0.02
	Mouthwash	89 (17.4)	63 (12.4)	152 (29.8)	
	Toothpick	71 (13.9)	35 (6.9)	106 (20.8)	
	Other	41 (8.0)	4 (0.8)	45 (8.8)	
	None	108 (21.1)	104 (20.4)	212 (41.5)	

Visit to dentist in past year	Yes	168 (32.9)	96 (18.8)	264 (51.7)	*χ* ^2^(1) = 8.41, *p* = 0.014
	No	124 (24.3)	115 (22.5)	239 (46.8)	

Reasons for visit to the dentist	Pain	90 (17.6)	64 (12.5)	154 (30.1)	*χ* ^2^(3) = 5.41, *p* = 1.41
	Oral diseases	12 (2.4)	9 (1.8)	21 (4.1)	
	Aesthetics	15 (2.9)	6 (1.2)	21 (4.1)	
	Routine check-up	56 (11.0)	21 (4.1)	77 (15.1)	

**Table 3:** Knowledge and awareness of school teachers regarding children's oral health.

Knowledge and awareness		English (%)	Marathi (%)	Total (%)	Chi square test
Dentition an individual has	Primary	3 (0.6)	2 (0.4)	5 (1.0)	*χ* ^2^(2) = 11.08, *p* = 0.004
	Permanent	139 (27.2)	66 (12.9)	205 (40.1)	
	Both	139 (27.2)	133 (26.0)	272 (53.2)	

Number of primary teeth	16	69 (13.5)	50 (9.8)	119 (23.3)	*χ* ^2^(3) = 15.94, *p* = 0.001
	20	154 (30.1)	78 (15.3)	232 (45.4)	
	28	57 (11.2)	68 (13.3)	125 (24.5)	
	32	2 (0.4)	0 (0)	2 (0.4)	

Importance of primary dentition	Yes	232 (45.4)	158 (30.9)	390 (76.3)	*χ* ^2^(2) = 2.72, *p* = 0.25
	No	30 (5.9)	22 (4.3)	52 (10.2)	
	Not sure	30 (5.9)	32 (6.3)	62 (12.2)	

Reason for child's first visit to dentist	Pain	30 (5.9)	24 (4.7)	54 (10.6)	*χ* ^2^(9) = 27.6, *p* = 0.01
	After six months	28 (5.5)	10 (2.0)	38 (7.5)	
	After primary teeth erupt	36 (7.1)	7 (1.4)	43 (8.5)	
	When primary teeth decay	32 (6.3)	17 (3.3)	49 (9.6)	
	When permanent teeth erupt	44 (8.6)	35 (6.9)	79 (15.5)	
	After 1-2 years	47 (9.2)	7 (1.4)	54 (10.6)	
	If there is any dental problem	43 (8.4)	23 (4.5)	66 (12.9)	
	When child starts chewing	6 (1.2)	4 (0.8)	10 (2.0)	
	To get dental information	5 (1.0)	7 (1.4)	12 (2.4)	
	Bad breath	2 (0.4)	1 (0.2)	3 (0.6)	

**Table 4:** Oral health knowledge and approaches of school teachers towards children.

Knowledge and approaches		English (%)	Marathi (%)	Total (%)	Chi square test
Number of times a child should brush	Once	3 (0.6)	2 (0.4)	5 (1.0)	*χ* ^2^(3) = 0.86, *p* = 0.83
	Twice	263 (51.5)	190 (37.2)	453 (88.7)	
	Thrice	29 (5.7)	22 (4.3)	51 (10.0)	

Awareness of brushing techniques for children	Yes	195 (38.2)	168 (32.9)	363 (71.1)	*χ* ^2^(1) = 6.4, *p* = 0.04
	No	91 (17.8)	45 (8.8)	136 (26.6)	

Tooth cleaning method used	Horizontal	63 (12.3)	29 (5.7)	92 (18.0)	*χ* ^2^(3) = 13.43, *p* = 0.04
	Vertical	41 (8.0)	34 (6.7)	75 (14.7)	
	Circular	103 (20.2)	108 (21.1)	211 (41.3)	
	Other	3 (0.6)	10 (2.0)	13 (2.6)	

Clean their mouth after snacking	Yes	160 (31.3)	131 (25.6)	291 (56.9)	*χ* ^2^(1) = 1.96, *p* = 0.16
	No	131 (25.6)	83 (16.2)	214 (41.8)	

Awareness of fluoridated toothpaste	Yes	153 (29.9)	71 (13.9)	224 (43.8)	*χ* ^2^(2) = 64.06, *p* = 0.000
	No	92 (18.0)	138 (27.0)	230 (45.0)	
	Not sure	49 (9.6)	5 (1.0)	54 (10.6)	

Awareness of school water fluoridation	Yes	19 (3.7)	18 (3.5)	37 (7.2)	*χ* ^2^(2) = 40.45, *p* = 0.00
	No	212 (41.5)	191 (37.4)	403 (78.9)	
	Not sure	62 (12.1)	4 (0.8)	66 (12.9)	

Does oral health affect general health	Yes	207 (40.5)	171 (33.5)	378 (74.0)	*χ* ^2^(2) = 8.56, *p* = 0.036
	No	35 (6.9)	17 (3.3)	52 (10.2)	
	Not sure	53 (10.4)	77 (15.1)	130 (25.5)	

Any oral health training	Yes	37 (7.2)	45 (8.8)	82 (16.0)	*χ* ^2^(2) = 7.58, *p* = 0.023
	No	251 (49.1)	164 (32.1)	415 (81.2)	
	Not sure	6 (1.2)	2 (0.4)	8 (1.6)	

Discussion with parents	Yes	130 (25.4)	94 (18.4)	224 (43.8)	*χ* ^2^(1) = 0.15, *p* = 0.90
	No	164 (32.1)	116 (22.7)	280 (54.8)	

**Table 5:** Differences in knowledge, practices and awareness between pre-primary and primary school teachers (English and Marathi medium).

Knowledge, practices, and awareness	English (%)		Chi square test	Marathi (%)		Chi square test
	Pre-primary school	Primary		Pre- primary school	Primary	
Level of education						
School	4 (6.3)	4 (1.7)	*χ* ^2^(3) = 7.59, *p* = 0.05	2 (8.3)	17 (9.0)	*χ* ^2^(3) = 21.3, *p* = 0.00
Higher secondary school	8 (12.7)	58 (24.8)		13 (54.2)	29 (15.3)	
Graduation	42 (66.7)	142 (60.7)		9 (37.1)	119 (63.0)	
After graduation	9 (14.3)	30 (12.8)		0 (0)	24 (12.7)	
Teaching experience						
<1 year	10 (15.9)	25 (10.7)	*χ* ^2^(2) = 3.4, *p* = 0.05	3 (12.5)	4 (2.1)	*χ* ^2^(2) = 9.7, *p* = 0.008
1–5 years	25 (39.7)	75 (32.1)		6 (25.0)	27 (14.3)	
>5 years	28 (44.4)	134 (57.3)		15 (62.5)	159 (83.6)	
Accessory tooth cleaning aids						
Floss	7 (11.1)	7 (3)	*χ* ^2^(4) = 10.17, *p* = 0.038	1 (4.2)	6 (3.2)	*χ* ^2^(4) = 10.25, *p* = 0.036
Mouthwash	22 (34.9)	67 (28.8)		6 (25)	57 (30.2)	
Toothpick	14 (22.2)	57 (24.5)		7 (29.2)	28 (14.8)	
Other	28 (44.4)	13 (5.6)		2 (8.3)	2 (1.1)	
None	19 (30.2)	89 (34.2)		8 (33.3)	96 (50.8)	
Dentition an individual has						
Primary	2 (3.3)	1 (0.5)	*χ* ^2^(2) = 6.21, *p* = 0.05	0 (0)	2 (1.1)	*χ* ^2^(2) = 5.97, *p* = 0.05
Permanent	37 (54.1)	102 (46.2)		12 (54.5)	54 (30.2)	
Both	26 (42.6)	113 (53.3)		10 (45.5)	123 (68.7)	
